# Kinetic and kinematic variables affecting trunk flexion during level walking in patients with lumbar spinal stenosis

**DOI:** 10.1371/journal.pone.0197228

**Published:** 2018-05-10

**Authors:** Tatsuya Igawa, Junji Katsuhira, Akira Hosaka, Kenta Uchikoshi, Shinichi Ishihara, Ko Matsudaira

**Affiliations:** 1 Department of Rehabilitation, International University of Health and Welfare Mita Hospital, Tokyo, Japan; 2 Faculty of Medical Technology, Department of Prosthetics and Orthotics and Assistive Technology, Niigata University of Health and Welfare, Niigata, Japan; 3 Department of Medical Research and Management for Musculoskeletal Pain, 22nd Century Medical and Research Center, The University of Tokyo, Tokyo, Japan; 4 Faculty of Health Science, Department of Locomotive Rehabilitation Science, Showa University, Tokyo, Japan; 5 Faculty of Health Science, Department of Assistive Technology Science, International University of Health and Welfare, Tokyo, Japan; 6 Spine and Spinal Cord Center, International University of Health and Welfare Mita Hospital, Tokyo, Japan; University of Münster, GERMANY

## Abstract

Lumbar spinal stenosis causes cauda equina and nerve root compression, resulting in neurological symptoms. Although trunk flexion during level walking may alleviate these symptoms by enabling spinal canal decompression, some patients do not use this strategy. We aimed to identify the kinetic and kinematic variables that affect trunk flexion in patients during level walking. Gait was recorded in 111 patients using a three-dimensional motion capture system and six force plates. From the data recorded, walking velocity, bilateral step length, cycle time, maximum trunk flexion angle, forward pelvic tilt angle, pelvic rotation angle, maximum and minimum joint angles, and moment and power of the lower limb were calculated. Then a step-wise multiple regression analysis was conducted to identify kinetic and kinematic variables affecting trunk flexion. The maximum hip extension angle (β = 0.416), maximum hip flexion moment (β = -0.348), and step length (β = 0.257) were identified as variables significantly affecting the trunk flexion angle. The coefficient of determination adjusted for the degree of freedom was 0.294 (p < 0.05). Our results suggest that patients with lumbar spinal stenosis choose one of two strategies to alleviate symptoms during walking. One strategy is gait with trunk flexion posture to increase step length and hip extension angle. The other strategy is gait with trunk upright posture to decrease step length and hip extension angle.

## Introduction

Spinal stenosis is caused by narrowing of the spinal canal or the various tunnels through which nerves and other structures communicate with that canal [1]. Symptomatic lumbar spinal stenosis (LSS) occurs with a prevalence of about 10% in the Japanese population over the age of 60 years [2]. A typical symptom is neurogenic intermittent claudication or increased pain in the legs with walking, which is caused by blockage of blood outflow from around the nerves. Symptoms such as these can generally be reduced in patients with LSS during lumbar spine flexion because this posture allows the vertebral foramen to open, thereby diminishing or eradicating neural compression [3–5]. As such, patients with LSS tend to be more comfortable walking uphill than downhill, and can walk further if they bend forward while walking [6]. Patients with LSS also show a wide-based gait with a short stride and cannot walk as they wish. For example, Yokogawa et al. reported that patients with LSS avoid leg pain by having increased physiological knee flexion immediately after foot-to-ground contact [7]. It has been also reported that many patients with LSS tend to walk with pronounced lumbar flexion at a slow speed, with abnormalities in gait style being immediately noticeable after the commencement of walking [8]. Postoperatively, however, trunk flexion after walking and the pelvic angle tend to decrease [9].

Although trunk flexion during level walking may effectively alleviate symptoms by enabling spinal canal decompression [3, 5, 6], some patients with LSS do not flex their trunk while walking. This suggests that there are multiple factors that affect the walking style in these patients. To our knowledge, there are no detailed reports examining the cause of trunk flexion using data from kinetic and kinematic gait analyses. Therefore, the purpose of this study was to identify kinematic and kinetic variables related to patients with LSS walking with trunk flexion or those walking in an upright position. We hypothesized that patients would respond by changing usage of the lower limb joints to match the inclination of their trunk, and those who do not flex their trunk would use another postural strategy to alleviate their symptoms.

## Materials and methods

All procedures were approved by the local ethics committee of the International University of Health and Welfare Mita Hospital and are consistent with the Declaration of Helsinki. Written informed consent was obtained from all patients before participation in this study.

### Patient recruitment

Patients with LSS at the International University of Health and Welfare Mita Hospital were enrolled in this study from June 2013 to August 2014. Patients were considered for enrollment if they were over 60 years of age and had LSS, as defined by Verbiest [10] (i.e., the presence of paresthesia or pain in the lower extremities, buttocks, perineum, or perianal region; and magnetic resonance imaging scans showing the presence of spinal canal stenosis that may explain the patient’s symptoms). In terms of disease pathogenesis, only patients with degenerative acquired stenosis (e.g., spondylolysis) were included; those with congenital, developmental, or post-traumatic LSS as well as those who had undergone spinal surgery were excluded. Other exclusion criteria were as follows: complications causing disorders that interfere with gait, such as those after cerebral infarction or myelopathy; diagnosis of lower extremity symptoms that are due to peripheral nerve diseases; Parkinson’s disease; previous surgery of the lower extremities; severe joint abnormalities in the lower limbs; cerebrovascular disease; and those receiving continuous infusion treatment as an inpatient.

Of the 373 patients who were screened, 116 patients (65 men and 51 women; mean age, 70.8 years), whose eligibility was guaranteed by third-party evaluation, were enrolled.

### Kinetic and kinematic data collection

Level walking was measured using a three-dimensional motion analysis system obtained by 10 MX cameras (Vicon Motion Systems, Oxford, UK) and six force plates (AMTI, Watertown, MA, USA) 1 or 2 days before spinal surgery at the patient’s selected speed and before the appearance of neurogenic claudication. The six force plates were arranged in three rows of two, and patients were instructed to step onto the right-hand plates with their right foot and onto the left-hand plates with their left foot. Kinematic and kinetic data were recorded at sample frequencies of 100 and 1000 Hz, respectively. The recorded kinematic and kinetic data were low-pass filtered using a second-order recursive Butterworth filter with respective cutoff frequencies of 6 and 18 Hz in accordance with the technique reported by Winter [11].

**Fig 1:**
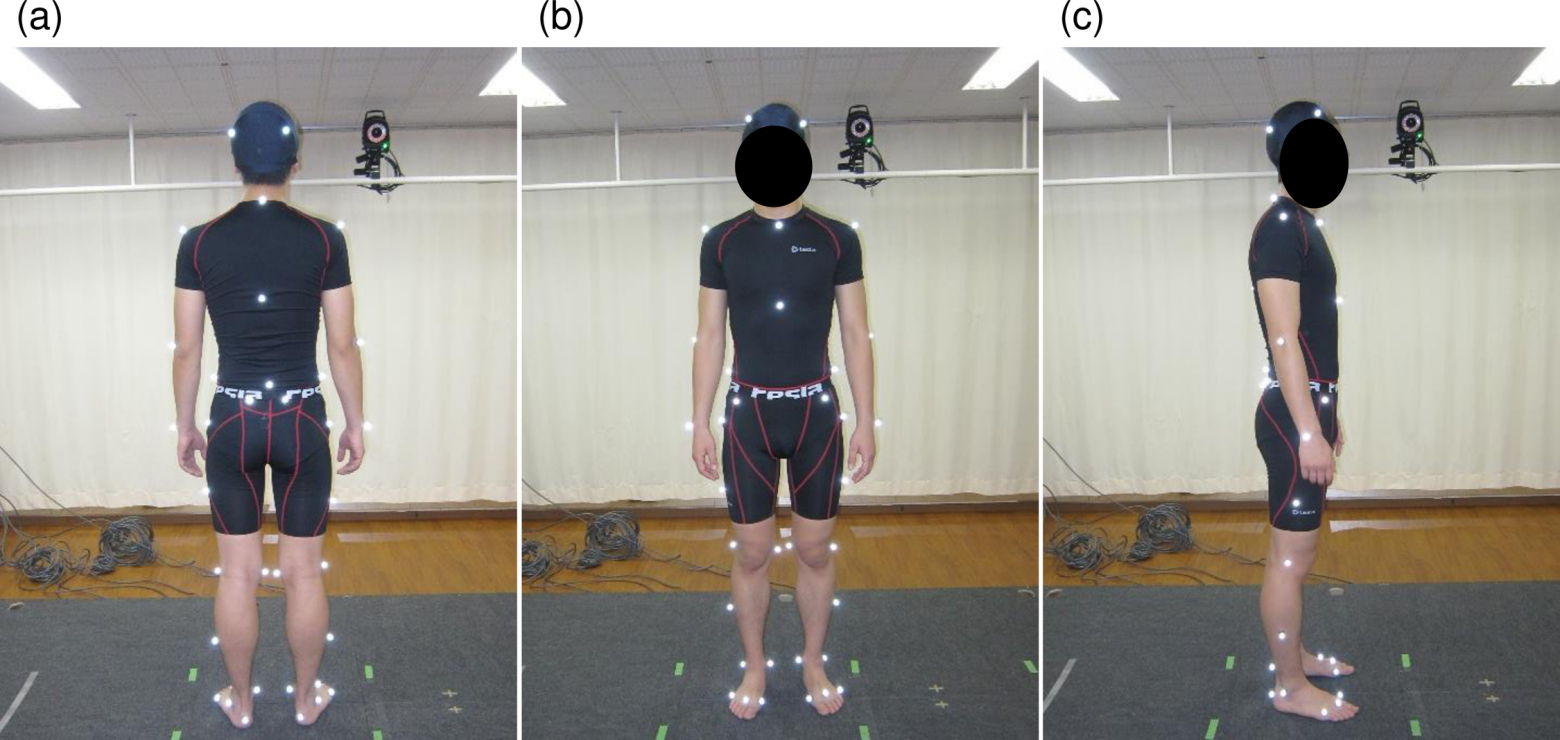
Forty-three reflective markers. (a) Posterior view, (b) anterior view, and (c) lateral view.

To construct anatomical coordinate systems for each body segment, 43 reflective markers with a diameter of 14 mm were used as anatomical markers (Fig 1A–1C). These were attached to the following landmarks, as per the Helen Hayes and Plug-in-gait Marker protocol [12]: the four points of the head; spinous process of the seventh cervical vertebra; manubrium sterni; xiphoid process; spinous process of the tenth thoracic vertebra; point between the fourth and fifth lumbar vertebrae; bilaterally on the acromion process; the lateral epicondyle; ulnar styloid process; iliac crest; anterior and posterior superior iliac spine; superior aspect of the greater trochanter; mediolateral knee; mid-point between the greater trochanter and lateral femoral condyles; medial and lateral malleoli; mid-point between the lateral knee joint line and lateral malleolus; first, second, and fifth metacarpophalangeal joints; and heel.

### Kinetic and kinematic data analysis

Joint kinematics and kinetics were analyzed using Visual3D analytical software (C-motion, Germantown, MD, USA). The trunk segment was defined using left and right iliac crest and shoulder markers. The seventh cervical vertebra, manubrium sterni, xiphoid process, and tenth thoracic vertebra markers were used as a cluster marker. The pelvic segment was defined using the CODA method from the anterior and posterior superior iliac spine markers. The thigh segment was defined using the mediolateral knee markers in addition to the hip joint center calculated by the CODA method. The superior aspect of the greater trochanter, lateral knee, and mid-point between the greater trochanter and lateral femoral condyle markers were used as cluster markers. The shank segment was defined using the mediolateral knee and ankle markers. The mediolateral knee and ankle markers were used as cluster markers. For the foot segment, we defined the ankle joint center and mediolateral ankle as the second metacarpophalangeal joint markers. The heel and the first, second, and fifth metacarpophalangeal joint markers were used as cluster markers. In the analysis, segments were regarded as rigid, and joint moments were calculated using a link segment model where segments were connected at nodal points. The ankle joint angle was calculated by the relative angle between the foot and shank segments. The hip, pelvis, and trunk angles were calculated by absolute angles of the thigh, pelvis, and trunk segments, respectively. All joint angles were calculated using a Cardan sequence of rotations in the orders of X, Y, and Z. To compute the joint moments, data regarding joint coordinates were added to the ground reaction force data, where the position of the center of mass, weight portion, and moment of inertia of each segment were used as parameters. From these data, the following were determined for the stance phase of one gait cycle in each patient: peak trunk and pelvis anterior tilt angle values; pelvis rotation angle; angle, moment, and joint power of the hip, knee, and ankle joints of both limbs; and spatiotemporal parameters. Spatiotemporal parameters included walking velocity, step length, and cycle time. Step length was normalized by body height, and joint moment and power were normalized by body mass. Gait parameters were determined for two gait cycles and then averaged to give a final value for each parameter.

### Clinical assessment

Patient symptoms were assessed using the low back pain section of the Japanese Orthopaedic Association Back Pain Evaluation Questionnaire (JOABPEQ), which evaluates several aspects of low back pain in patients. The reliability of this questionnaire has been examined and is now available for clinical use [13]. The low back pain score includes results from five categories (low back pain, lumbar function, walking ability, social life function, and mental health) that have been selected from the Roland Morris Disability Questionnaire and Short Form 36 and visual analog scale [14, 15]. In addition, we questioned the degree of lower limb pain during gait measurements using the visual analog scale. The low back pain category of the JOABPEQ and degree of lower limb pain during gait measurement were used for statistical analysis.

### Statistical analysis

To determine the relationship between the anterior trunk flexion angle and the other measured parameters, including kinematic and kinetic variables and pain-related score, the Pearson’s correlation coefficients were calculated and a stepwise multiple linear regression analysis (using the stepwise option) was performed using SPSS software, version 21 for Windows (IBM Corp., Armonk, NY, USA). A single regression significance level of p < 0.05 was fixed for entry into the multiple regression analysis. The proportion of total variance in the dependent variable, accounted for by the independent variables, is reported as the square of the correlation coefficient (R^2^). The post hoc power analysis was performed using G*Power 3.1 (Heinrich Heine University, Düsseldorf, Germany) [16] to determine whether the sample size in multiple regression analysis had sufficient detection power.

## Results

**Fig 2:**
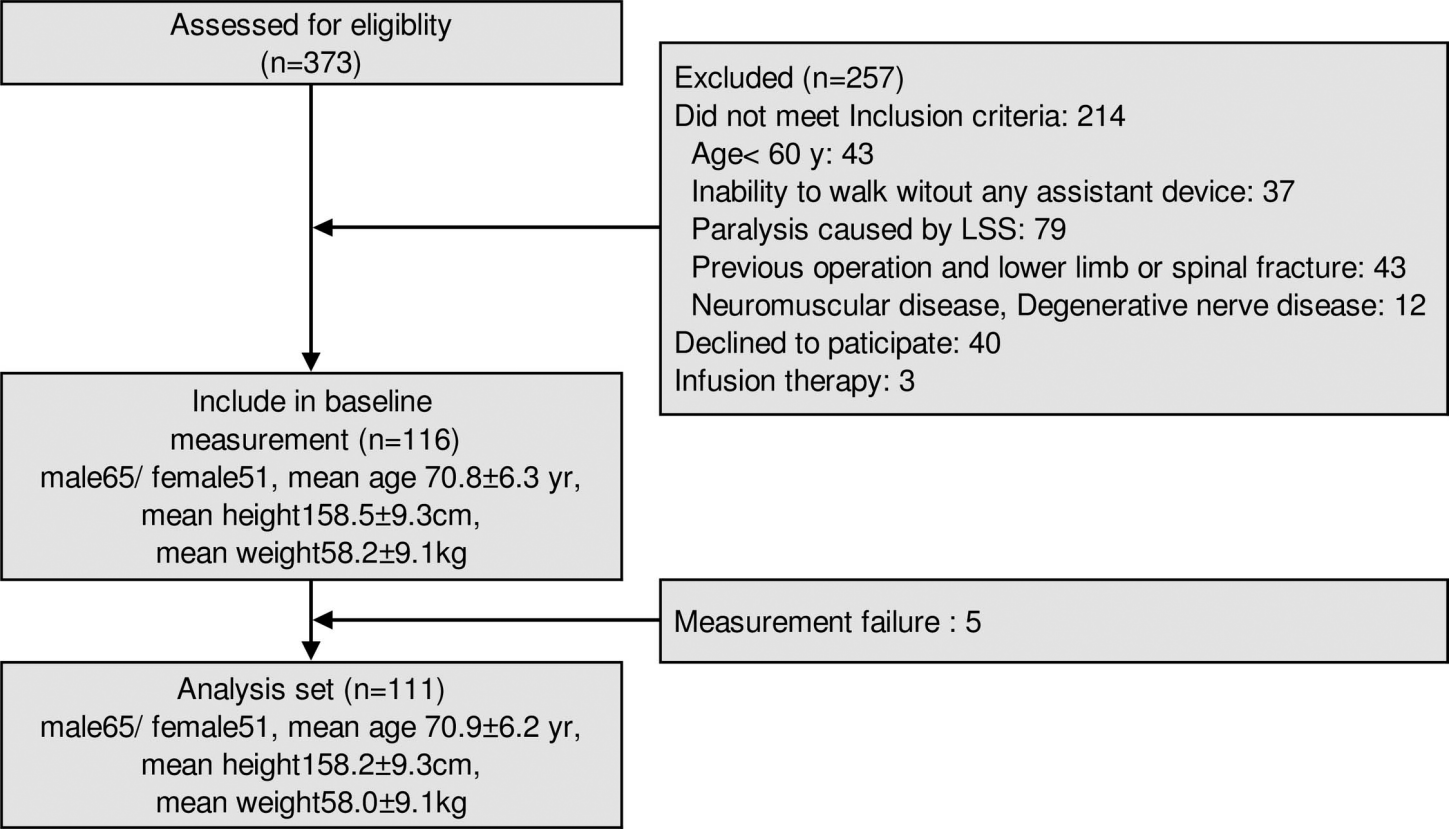
Study follow diagram. FRF, floor reaction force.

Characteristics of the study population are shown in Fig 2 and Table 1, and the results of the correlation and regression analyses are shown in Tables 2 and 3, respectively. In Pearson’s correlation coefficient analyses, step length, maximum forward pelvis tilt angle, maximum hip and knee flexion angle, maximum hip and knee extension angle, maximum hip flexion and ankle dorsi flexion moment, and minimum hip power were all significantly correlated with the maximum trunk flexion angle. In our stepwise multiple linear regression analysis with maximum trunk flexion angle as the dependent variable, the model found to best describe the variance was as follows.

**Table 1:** Patients’ clinical characteristics.

Stenotic part		Number of patients with LSS	with DH	with DS	with IS	JOABPEQ score (points)					VAS (mm)		
						BP	LF	WA	SF	MH	BP	LP	LN
**one level**	**L3/4**	1	1	0	0	46.8 (33.8)	61.4 (20.3)	38.5 (27.8)	47.8 (24.5)	47.3 (11.2)	47.7 (26.1)	64.9 (33.9)	58.0 (33.2)
	**L4/5**	7	7	2	0								
	**L5/1**	3	3	1	0								
**two level**	**L2/3 and L3/4**	1	1	0	0	51.6 (32.2)	68.5 (20.6)	39.0 (25.7)	45.0 (20.7)	48.0 (14.3)	50.1 (25.4)	65.6 (25.2)	60.4 (25.7)
	**L3/4 and L4/5**	39	39	4	7								
	**L4/5 and L5/1**	7	7	0	1								
**multi level**	**L2/3, L3/4 and L4/5**	21	21	2	5	55.6 (30.3)	72.8 (22.4)	35.7 (27.0)	45.1 (19.4)	49.1 (16.8)	47.7 (25.5)	61.5 (22.7)	61.4 (27.8)
	**L3/4, L4/5 and L5/1**	13	13	2	2								
	**L1/2, L2/3, L3/4 and L4/5**	3	3	0	1								
	**L2/3, L3/4, L4/5 and L5/S1**	13	13	3	3								
	**L1/2, L2/3, L3/4, L4/5 and L5/S1**	3	3	0	0								

LSS, lumbar spinal stenosis; DH, disc herniation; DS, degenerative spondylolisthesis; IS, isthmic spondylolisthesis; JOABPEQ, Japanese orthopedic association back pain evaluation questionnaire; VAS, visual analog scale; BP, low back pain; LF, lumbar function; WA, walking ability; SF, social life function; MH, mental health; LP, leg pain; LN, leg numbness; Values for JOABPEQ and VAS are represented mean and standard deviation.

**Table 2:** Correlation analysis of variables possibly associated with the maximum trunk flexion angle (n = 222).

	Mean (SD)		Pearson's Correlation Coefficient	P Value
Correlation Coefficient
VAS (mm)	44.5	(30.8)	-0.07	0.29
JOABPEQ (point)	37.3	(27.1)	0.12	0.08
Step length (m)	0.5	(0.1)	0.17	0.01
Velocity (m/s)	0.8	(0.2)	0.12	0.06
One gait cycle time (s)	1.1	(0.2)	0.08	0.24
Maximum pelvis rotation angle (deg)	3.5	(3.1)	0.01	0.85
Maximum pelvis anterior tilt angle (deg)	-9.6	(6.1)	0.29	p<0.01
Maximum hip flexion angle (deg)	20.2	(3.7)	0.27	p<0.01
Maximum hip extension angle (deg)	13.6	(5.3)	0.47	p<0.01
Maximum knee flexion angle (deg)	39.8	(5.8)	0.33	p<0.01
Maximum knee extension angle (deg)	-4.6	(5.6)	0.33	p<0.01
Maximum ankle dorsi flexion angle (deg)	15.6	(3.6)	0.09	0.18
Maximum ankle plantar flexion angle (deg)	7.9	(4.5)	0.07	0.30
Maximum hip flexion moment (Nm/kg)	0.6	(0.2)	0.44	p<0.01
Maximum hip extension moment (Nm/kg)	0.5	(0.2)	-0.11	0.11
Maximum knee flexion moment (Nm/kg)	0.2	(0.1)	0.05	0.43
Maximum knee extension moment (Nm/kg)	0.3	(0.1)	0.06	0.35
Maximum ankle dorsi flexion moment (Nm/kg)	0.1	(0.1)	0.08	0.22
Maximum ankle plantar flexion moment (Nm/kg)	1.2	(0.2)	0.20	p<0.01
Maximum hip power (W/kg)	0.7	(0.3)	0.06	0.36
Minimum hip power (W/kg)	-0.4	(0.2)	-0.38	p<0.01
Maximum knee power (W/kg)	0.3	(0.2)	0.21	p<0.01
Minimum knee power (W/kg)	-0.8	(0.4)	-0.08	0.24
Maximum ankle power (W/kg)	2.3	(0.8)	0.07	0.27
Minimum ankle power (W/kg)	-0.6	(0.2)	0.00	0.99

VAS, visual analog scale; JOABPEQ, Japanese orthopedic association back pain evaluation questionnaire.

**Table 3:** Multiple linear regression analysis of data of patients with lumbar spinal stenosis (n = 222).

Coefficients									
	Unstandardized Coefficients		Standardized			95% Confidence Interval for B		Collinearity Statistics	
	B	Std. Error	Beta	T	p-value	Lowder Bound	Upper Bound	Tolerance	VIF
**(Constant)**	1.61	1.65		0.97	0.33	-1.64	4.86		
**Maximum hip joint extension angle**	0.36	0.06	0.42	5.59	p<0.001	0.23	0.49	0.58	1.73
**Maximum hip joint flexion moment**	-8.01	1.68	-0.35	-4.78	p<0.001	-11.31	-4.70	0.60	1.67
**Step length**	15.25	4.34	0.26	3.51	p<0.001	6.69	23.81	0.60	1.68

Total model r2 = 0.29, F statistic = 31.74 (p<0.01)

Dependent variable: maximum trunk flexion angle

$$\begin{array}{l} Maximum\;anterior\;trunk\;flexion\;angle\;=\\ \left(0.36\times maximum\;hip\;extension\;angle\right)\;–\left(8.01\times maximum\;hip\;flexion\;moment\right)\;+\\ \left(15.25\times step\;length\right)\;+\;1.607 \end{array}$$

A significant regression equation was found (F (3, 218) = 31.736, p <.000) with an R^2^ of 0.29, thus explaining 29% of the total variance in the maximum trunk flexion angle (Table 3). The degree of leg pain and gait disturbance, as assessed by the JOABPEQ, did not significantly contribute to the model. As a result of the post hoc power analysis (effect size f^2^ = 0.408, total sample size = 111, total number of tested predictors = 4, total number of predictors = 10, p < 0.05), power (1 - β) = 1.000, sufficient detection power was obtained.

## Discussion

**Fig 3:**
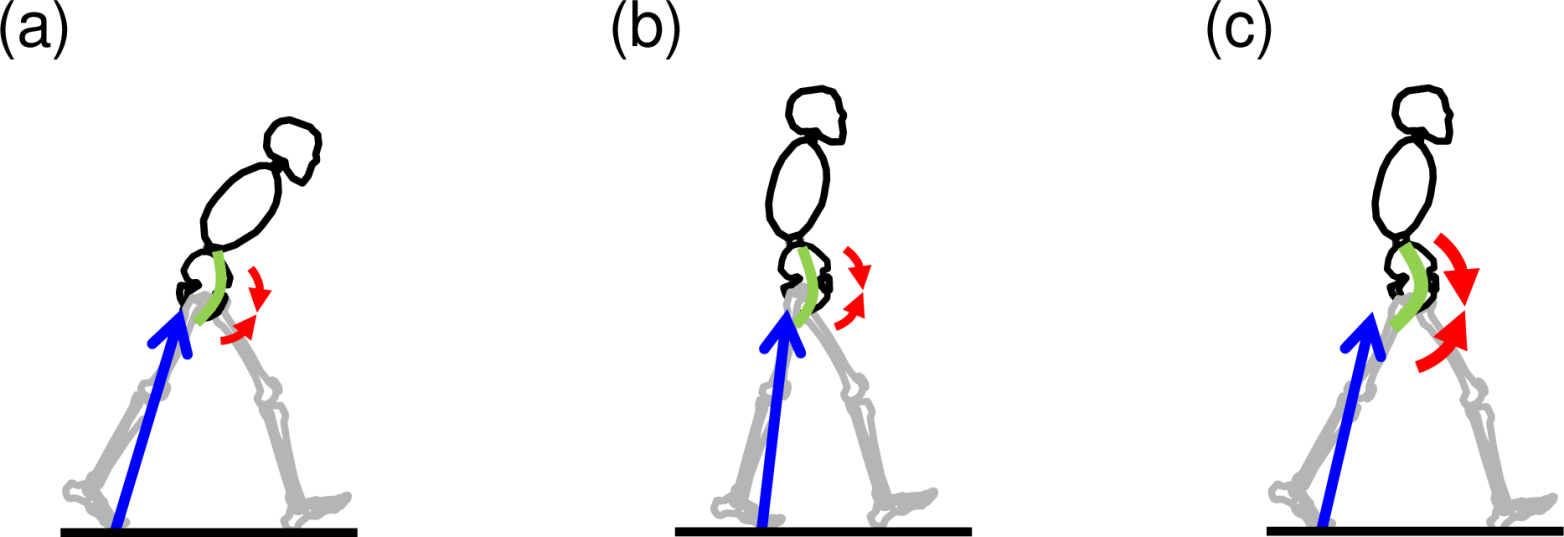
Two strategies used by patients with lumbar spinal stenosis to alleviate symptoms. (a) Trunk flexion posture with an increased step length and hip extension angle. (b) Trunk upright posture with a decreased step length and hip extension angle. (c) Ideal walking posture of healthy people. Red arrows indicate the ground reaction force vector, blue arrow is the hip flexion moment, and green arc is the psoas major.

Several previous studies have analyzed the relationship between the trunk flexion posture and thoracic lumbar vertebrae and pelvic parameters by means of radiographic measurement [17, 18]. However, these studies evaluated static alignment, and factors affecting adoption of the trunk flexion posture during dynamic walking have yet to be investigated. This study is the first to analyze factors related to trunk flexion using kinetic and kinematic data obtained from a motion analysis system and a pain evaluation questionnaire. In addition to stride, only the hip joint angle and hip joint moment were related to the trunk flexion posture. Specifically, the stride of community-dwelling elderly persons becomes shorter when walking with the trunk in flexion [19]. In the present study, however, it was shown that patients with LSS used one of two different strategies: 1) gait with trunk flexion posture to increase step length and the hip extension angle, and 2) gait with trunk upright posture to decrease step length and hip extension angle. The reason why patients with LSS used these strategies was to avoid using hip flexion moment generated by the hip flexor muscle groups, e.g., the psoas major, in order to increase lumbar lordosis. Therefore, our results suggest that patients with LSS might choose one of those two strategies to alleviate their symptoms during walking. The first strategy (Fig 3A) involves increased trunk flexion, which decreases the hip flexion moment while using a strategy to increase stride and the hip extension angle so the flexed upper trunk does not pass the ground reaction force (GRF) vector of the posterior hip joint center. The second strategy (Fig 3B and 3C) instead involves maintenance of the trunk upright position. In this posture, the GRF vector easily passes the anterior hip joint center; therefore, hip flexion moment is likely to increase, requiring patients to avoid increasing their step length and hip extension angle.

Both strategies may contribute to decreased lumbar lordosis during walking by affecting the activity of the hip flexor muscles. However, although many studies have been conducted to evaluate these muscle activities including of the psoas major, the role of muscle activities is not yet definitive [20–22]. The most recent model analysis revealed that the psoas major has a lumbar extension effect [23]. It has been also reported that tension of the large psoas muscle and lumbar lordosis are increased when the hip joint extension angle peaks in the late stance phase of walking [24, 25]. Furthermore, intermittent claudication can be induced during this phase if lumbar lordosis is enhanced by psoas major muscle action [3, 4]. For this reason, it can be assumed that patients with LSS who use the trunk upright posture while walking can reduce lumbar lordosis and alleviate their symptoms by reducing step length and the hip joint extension angle to minimize psoas major muscle tension. On the other hand, some patients with LSS reduce lumbar lordosis by adopting the trunk flexion posture while walking. Since tension of the large psoas muscle decreases when the trunk is bent forward, patients who use this posture are able to walk with longer strides and greater hip extension angles than those who use the upright posture.

These strategies also affect epidural pressure. It has been reported that epidural pressure is higher in patients with LSS than in healthy subjects, and higher in the upright than in the lumbar flexion posture [26–28]. Additionally, it has been shown that epidural pressure changes are complicated in symptom-induced postural dependence, meaning that epidural pressure and the local pressure of the intervertebral foramen are related to posture. Furthermore, it has been reported that epidural pressure is decreased when walking with short strides [26]. Whether patients choose the trunk flexion or trunk upright posture may thus be related to these epidural pressure changes. Fluctuation of epidural pressure during the gait cycle is also similar to that of the hip flexion moment, as measured in this study, with peaks in these variables occurring at the same time. Therefore, hip flexion moment during walking may be a parameter that can be used to indirectly estimate epidural pressure fluctuation. It may also be an important factor to consider for symptom reduction. In particular, it can be inferred that LSS symptoms worsen when hip flexion moment is significantly increased by walking with the trunk in the upright posture and with an increased stride.

For this reason, we believe that it is desirable to select one of the posture types and instruct patients to walk in that manner to alleviate their symptoms. It has been reported that posture after the occurrence of intermittent claudication is characterized by an increased anterior trunk flexion angle [9]. Therefore, it can be presumed that the load on the lumbar back muscles is increased when intermittent claudication appears in patients walking with the trunk flexion posture. Hence, we recommend that these patients instead walk using the trunk upright posture with decreased step length. By walking in the trunk upright posture to avoid symptoms for as long as possible, patients with LSS can increase the amount of physical activity they perform. Since engaging in exercise, such as walking and cycling, contributes to the maintenance of health in elderly individuals [29], using this strategy can in turn enable patients to prolong a healthy life.

Despite the insights provided by this study, there are several limitations to consider. First, to clarify the relationship between tension of the psoas major and epidural pressure, further investigation is necessary. Second, since subjects of this study included patients at only our hospital, the presence of selection bias is possible. Third, sagittal alignment parameters such as spinal deformation and the lumbar lordosis angle may also be involved in the trunk flexion posture during walking, but they were not investigated in our study. Future multicenter, collaborative studies that include the measurement of sagittal alignment using radiographic parameters are needed.

## Conclusions

In our study, the maximum hip extension angle, maximum hip flexion moment, and step length were identified as variables affecting trunk flexion in patients with LSS during walking. This suggests that patients with LSS might choose one of two strategies to alleviate their symptoms during walking. One strategy is gait with trunk flexion posture to increase step length and the hip extension angle. The other strategy is gait with trunk upright posture to decrease step length and the hip extension angle.

## Supporting information

S1 Table Data of the kinetic and kinematic variables. (XLSX)
